# Supplementary material for: Identification of photoperiod-induced specific miRNAs in the adrenal glands of Sunite sheep (Ovis aries)
Source: Front Vet Sci. 2022 Jul 22;9:888207. doi: 10.3389/fvets.2022.888207 (PMC9354845; doi:10.3389/fvets.2022.888207)
Supplement: Supplementary file 1 [file Data_Sheet_1.ZIP › Supplementary file/miRDeep2 file.zip!/miRDeep2 file/Novel_Structure/SPLP42A3/Novel_570.pdf]

Provisional ID	:	NC_019479.2_33603
Score total	:	1.8
Score for star read(s)	:	-1.3
Score for read counts	:	0
Score for mfe	:	1.5
Score for randfold	:	1.6
Score for cons. seed	:	
Total read count	:	393
Mature read count	:	393
Loop read count	:	0
Star read count	:	0

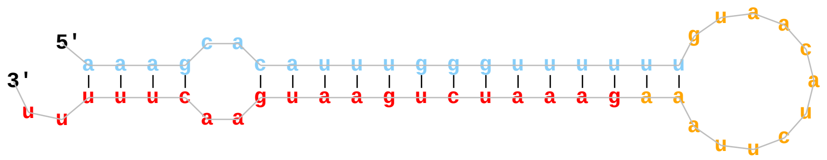

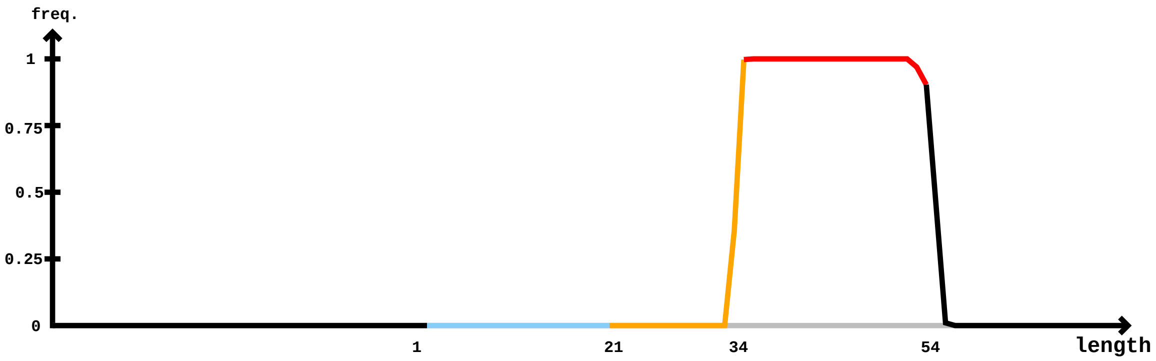

Star Mature

```
5'- accaaaauuacagaggcuaauuuauuggguuggcuaaaaagcacauuuggguuuuuuguaacaucuuaaagaaaucugaaugaacuuuuugggcaacccaauacauucua   -3'   exp
    ......................((((((((((.((((((((..((((((((((((((...........))))))))))))))..)))))))).))))))))))).......    reads    mm    sample
    .....................................................................aAaaaucugaaugaacuuu......................    12       1     seq
    .....................................................................aAaaaucugaaugaacuuuu.....................    26       1     seq
    .....................................................................aAaaaucugaaugaacuuuuu....................    78       1     seq
    .....................................................................aAaaaucugaaugaacuuuuug...................    24       1     seq
    ......................................................................gaaaucugaaugaacuuuuu....................    1        0     seq
    ......................................................................Aaaaucugaaugaacuuuuu....................    247      1     seq
    ......................................................................gaaaCcugaaugaacuuuuug...................    1        1     seq
    ......................................................................gaaaucugaaugaacuuuuugg..................    1        0     seq
    ......................................................................gaaaCcugaaugaacuuuuugg..................    2        1     seq
    .......................................................................aaaucugaaugaacuuuuugC..................    1        1     seq
```
